# Can Exercise Reduce the Autonomic Dysfunction of Patients With Cancer and Its Survivors? A Systematic Review and Meta-Analysis

**DOI:** 10.3389/fpsyg.2021.712823

**Published:** 2021-08-24

**Authors:** Ana Myriam Lavín-Pérez, Daniel Collado-Mateo, Xián Mayo, Gary Liguori, Liam Humphreys, Alfonso Jiménez

**Affiliations:** ^1^PhD International School, Program of Epidemiology and Public Health (Interuniversity), Rey Juan Carlos University, Móstoles, Spain; ^2^Centre for Sport Studies, Rey Juan Carlos University, Fuenlabrada, Spain; ^3^GO fitLAB, Ingesport, Madrid, Spain; ^4^College of Health Sciences, University of Rhode Island, Kingston, NY, United States; ^5^Advanced Wellbeing Research Centre, College of Health, Wellbeing and Life Sciences, Sheffield Hallam University, Sheffield, United Kingdom

**Keywords:** autonomic modulation, exercise programs, cardiovascular dysfunction, oncology, heart rate variability

## Abstract

**Background:** Cancer therapies have increased patient survival rates, but side effects such as cardiotoxicity and neurotoxicity can lead to autonomic nervous and cardiovascular system dysfunction. This would result in a decrease in parasympathetic activity and the enhancement of sympathetic activity. Heart rate variability (HRV), which reflects autonomic modulation, is a valuable physiological tool since it correlates with cancer-related fatigue, stress, depression, and mortality in patients with cancer.

**Objective:** This study aimed to analyze the effects of exercise programs on the autonomic modulation, measured by the HRV of patients with cancer and its survivors.

**Methods:** The Preferred Reporting Items for Systematic Reviews and Meta-Analyses (PRISMA) guidelines were followed, and the quality of the articles was assessed with the Physiotherapy Evidence Database (PEDro) scale. The meta-analysis statistic procedure was performed by using RevMan software version 5.3.

**Results:** From the 252 articles found, six studies were included in the review involving 272 participants aged 30–75 years. Exercise programs had a mean length of 10.4 ± 4.6 weeks, a frequency of 3 ± 1.4 days/week, and a mean duration of 78 ± 23.9 min. In time-domain HRV measures, exercise may increase in the SD of normal-to-normal intervals [*p* < 0.00001, with a mean difference (MD) of 12.79 ms from 9.03 to 16.55] and a decreased root mean square of successive R–R interval differences (*p* = 0.002, with an MD of 13.08 ms from 4.90 to 21.27) in comparison with control groups (CG). The frequency-domain data reveal that the exercise group (EG) improve significantly more than the CGs in low frequency [absolute power: *p* < 0.0001, with a standardized mean difference (SMD) of 0.97 from 0.61 to 1.34; relative power: *p* = 0.04, with an MD = −7.70 from −15.4 to −0.36], high-frequency [absolute power: *p* = 0.001, with a SMD of 1.49 from 0.32 to 2.66; relative power: *p* = 0.04, with an MD of 8.00 normalized units (n.u.) from 0.20 to 15.80], and low-to-high frequency ratio (*p* = 0.007 with an MD of −0.32 from −0.55 to −0.09).

**Conclusion:** Exercise programs could lead to positive effects on the autonomic modulation of patients with cancer and its survivors. More beneficial changes may occur with resistance and endurance workouts. However, due to the low number of interventions performed, further research is needed to substantiate the findings and to provide additional insights regarding the exercise intensity required to increase the autonomic modulation of the patient.

## Introduction

Modern advances in cancer therapy have resulted in increased survival rates. However, patients are frequently affected by various negative side effects (Miller et al., 2019). In particular, both the autonomic control impairment and the increased risk of cardiovascular disease (Lakoski et al., 2015) are suffered by ~80% (Coumbe and Groarke, 2018) and 42% (Chen et al., 2012) of the patients, respectively. The autonomic nervous system (ANS) is the main homeostatic regulatory system of the body, involved in the etiology and the clinical course of cancer therapies (Simó et al., 2018). Chemotherapy and radiotherapy cause cardiotoxicity (Scott et al., 2014) and neurotoxicity (Park et al., 2013), thereby affecting ANS and cardiovascular function. The cardiovascular ANS needs to be controlled during the cancer phases to regulate levels of autonomic dysfunction of patients (Walsh and Nelson, 2002).

Heart rate variability (HRV) is a noninvasive tool to evaluate the autonomic modulation of the sinus node in healthy, cardiac, and noncardiac disease populations (Lombardi and Stein, 2011). In patients with cancer, HRV is a useful, effective, and more practical method to assess autonomic dysfunction than other validated methods, which are more complex to apply (Guo et al., 2013). HRV measures provide a multidimensional register of autonomic modulation, including the sympathetic and parasympathetic modulation of cardiac function (Arab et al., 2016), which is notably modified in patients with cancer compared with the healthy population. Studies including wide sample sizes have found significantly lower values in the root mean square of successive R–R interval measures (RMSSD), representing the parasympathetic activity, and the SD of the interbeat interval of normal sinus beats (SDNN) (i.e., overall HRV measure) of patients with cancer compared with the healthy population (De Couck and Gidron, 2013; Bijoor et al., 2016). Cancer-treating drug therapies induce cardiac abnormalities, such as heart failure, myocardial ischemia, myocarditis, hypertension, or arrhythmias, among others (Chang et al., 2017), detected with HRV even with normal systolic left ventricular function (Tjeerdsma et al., 1999) with an overactivation of the sympathetic nervous system (SNS) and a decrease in the parasympathetic nervous system (PNS) activity (Coumbe and Groarke, 2018). Consequently, this imbalance, produced by cancer drugs, may stimulate the hypothalamic-pituitary-adrenal axis and the endocannabinoid and renin-angiotensin-aldosterone systems producing an increase in oxidative stress, chronic inflammation, and atherosclerosis with a reduction in vasodilation, which affects the health of patients (Lakoski et al., 2015). Several types of research have focused on the relationship between the global HRV modifications and the health of patients with cancer obtaining an inverse correlation with cancer-related fatigue (Fagundes et al., 2011) and depression (Giese-Davis et al., 2006). Findings also show the role of HRV to predict survivorship being higher when the vagal nerve activation is stimulated (Zhou et al., 2016).

Palma et al. (2020) and Dias Reis et al. (2017) published a systematic review of supportive therapy modalities that have been developed to reduce the HRV results. They found interventions based on music therapy, traditional Chinese medicine-related treatments, exercise, relaxation, and myofascial release techniques and concluded that HRV seemed to be a safe and easily applicable method to assess cancer-related autonomic dysfunction (Palma et al., 2020). However, not only the consequences of cancer therapy need to be considered to improve HRV but also the lifestyles of patients can influence it negatively (Scott et al., 2014). Thus, a multifactorial intervention that could modify the physiological changes mentioned earlier and other health and psychological parameters is crucial to globally benefit the patients with cancer.

Exercise programs appear to promote physiological changes leading to reduce the decline of the autonomic modulation and improving its levels during (Mostarda et al., 2017) and after cancer treatments (Shin et al., 2016). Scott et al. (2014) approximated the benefits of aerobic exercise training to the autonomic modulation of patients with cancer stating that it may decrease sympathetic tone and increase vagal tone by the influence of exercise in attenuate cardiovascular abnormalities as heart failure or coronary artery disease. Aerobic exercise modifies the renin-angiotensin-aldosterone system (Scott et al., 2014) and stimulates the hypothalamic-pituitary-adrenal axis suppressing angiotensin II expression, which promotes the sympathetic activity of the ANS (Routledge et al., 2010). Consequently, the stimulation of the hypothalamic-pituitary-adrenal axis and the endocannabinoid and renin-angiotensin-aldosterone systems is produced (Arab et al., 2016). Exercise also upregulates nitric oxide promoting vasodilation (Kingwell, 2000; Scott et al., 2014) reduces reactive oxygen species induced by chemotherapy toxicity (Scott et al., 2014). Moreover, regarding the remaining physiological changes mentioned earlier, exercise can also decrease chronic inflammation (Gleeson et al., 2011), improving the immune system and the stimulation of natural killer cells (Khosravi et al., 2019). Exercise can also reduce body mass index (BMI) (Thomas et al., 2017), which may be correlated to an HRV increase (Arab et al., 2016). Although exercise may have the potential role to increase HRV, the results presented by the different interventions developed are controversial considering all the variables inside HRV. In this way, this systematic review and meta-analysis aimed to evaluate the effects of exercise interventions on the autonomic function of patients with cancer and its survivors analyzing the measures involved.

## Methods

The Preferred Reporting Items for Systematic Reviews and Meta-Analyses (PRISMA) guidelines have been followed to develop the systematic review (Liberati et al., 2009). Before the data extraction was performed, the study was registered in the International Prospective Register of Systematic Reviews (PROSPERO) by the following identification number: CRD42020191041.

### Search Strategy

The databases employed for searching the articles were Web of Science (including studies indexed in the KCI-Korean Journal Database, MEDLINE, Russian Science Citation Index, SciELO Citation Index, and PubMed (MEDLINE). The terms used for the search were as follows: cancer and neoplasms, separated by OR; and HRV, autonomous nervous function, autonomous nervous system, separated by OR; and exercise, training, physical activity, also separated by OR. The only filter employed was the requirement of being articles written in English or Spanish. The search was carried out from April 2020 to June 2020.

The articles found were included if they fulfilled the following criteria: (1) the target population was patients with cancer or survivors, (2) the program involves any physical exercise, (3) the investigation assesses and reports any HRV variable, (4) the study includes at least one control group (CG) whose results are compared with an exercise group (EG). Moreover, the following exclusion criteria were set: (1) the article was a review, letter to the editor, conference abstract, case report, or study protocol, (2) the study did not evaluate the HRV variables directly after the intervention, and (3) the studies were completely written in a language different from English or Spanish.

### Risk of Bias Assessment

The analysis of the risk of bias was performed using the Physiotherapy Evidence Database (PEDro) scale, known as a valid and reliable instrument to assess eligibility, allocation to groups, blinding of allocation, and comparison between groups at baseline and its outcomes (Maher et al., 2003). The leading reasons for its selection were due to it being the most used in the scientific area of the Sport Sciences for Health and it is a specific tool focused on physical therapies (Moseley et al., 2020).

### Data Extraction

#### Participants, Interventions, Comparisons, and Study Designs Information

The main information of the articles is reported in the tables and figures in the article and the supplementary data. Regarding participants, CG and EG baseline parameters were extracted, such as sample size, mean age, BMI, physical activity level, cancer type, stage, type of treatment, and timing. The intervention characteristics included the length of the program, duration of each session, weekly frequency, exercise description, intensity, progression, and adherence. The intervention of the comparison group was also extracted.

#### HRV Outcome Measurements

Interventions included an HRV assessment before and after the intervention. For consistency in measures, participants were laid in a supine position (Niederer et al., 2013; Dias Reis et al., 2017; Zhou et al., 2018) or seated in chairs (Shin et al., 2016; Lee et al., 2018). HRV was recorded for 5 min of adequate stationary sign (Niederer et al., 2013; Lee et al., 2018; Zhou et al., 2018) with Polar S810 (Niederer et al., 2013), CANS 3000 (Laxtha, Daejeon, Korea) (Shin et al., 2016), or CheckMyHeart Handheld HRV (Lee et al., 2018) by using ECG (Zhou et al., 2018). The spectral analysis from the artifact-free data was performed by the fast Fourier transformation with the Kubios HRV Analysis software (Niederer et al., 2013; Dias Reis et al., 2017; Mostarda et al., 2017; Zhou et al., 2018) or AcqKnowledge (Zhou et al., 2018). The results of this review include two HRV variables in the time domain, namely, SDNN and RMSSD. In the frequency domain, the low-frequency (LF) band, the high-frequency (HF) band, the ratio of LF-to-HF (LF/HF), and the total power (TP), calculated as the sum of the energy in very-low-frequency (VLF), LF, and HF bands, were extracted (Shaffer and Ginsberg, 2017). The frequency-domain data were reported in absolute units by using milliseconds squared (ms^2^) or normalized units (n.u.) obtained by diving the result between the TP and multiplied to 100 [i.e., LF or HF/(TP–VLF) × 100] (Task Force of the European Society of Cardiology the North American Society of Pacing Electrophysiology, 1996). SDNN and RMSSD were measured in 1/1,000 s (milliseconds) (Shin et al., 2016), and HF was recorded from 0.15 to 0.4 Hz and LF from 0.04 to 0.15 Hz (**Table 3**) (Niederer et al., 2013; Shin et al., 2016; Dias Reis et al., 2017; Mostarda et al., 2017; Zhou et al., 2018).

### Statistical Analysis

The analysis was performed with the post-intervention means and SDs of the EG and the CG collected from the articles. The meta-analysis statistical tool used was the Review Manager Software (RevMan software version 5.3) (RevMan, 2014). The selected method was the inverse variance with random effects and a 95% CI (Schmidt et al., 2009). Additionally, the I^2^ model was used to calculate the heterogeneity of the results. The results were presented with a standardized mean difference (SMD) when the same variable was measured in different units (i.e., ms^2^ and log ms^2^) as occurred with the absolute power variables of HF and LF. In contrast, the mean difference (MD) was used when all studies assessed the variable using the same units (n.u. or ms). According to the *Cochrane Handbook for Systematic Reviews of Intervention*, the SMD effects were interpreted as small with results <0.4, moderate from 0.4 to 0.7, and large >0.7 (Higgins and Green, 2011).

## Results

### Study Selection

**Figure 1:**
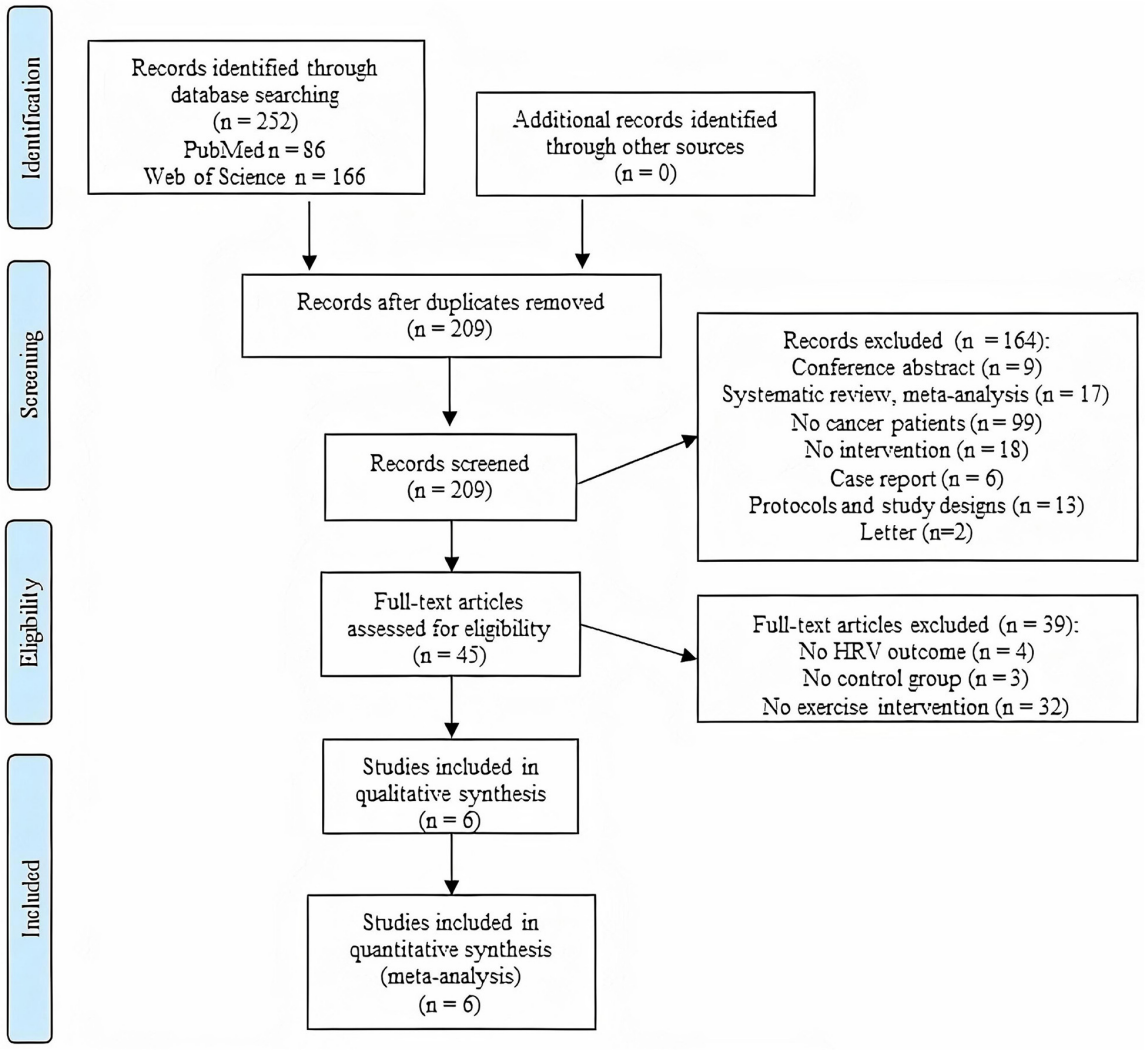
Study flow diagram following the preferred reporting items for systematic reviews and meta-analyses guidelines.

About 252 articles were identified in Web of Science (*n* = 166) and PubMed (*n* = 86) scientific databases. Figure 1 shows the flow diagram where 209 articles were analyzed after removing the duplicates. Two hundred and three articles were excluded after reading the title or the abstract (*n* = 164) or after the full-text evaluation (*n* = 39). Therefore, both the systematic review and the meta-analysis were developed with six studies published between 2012 and 2018.

### Risk of Bias

In the PEDro scale, as Table 1 shows the mean score obtained was 5, and the external validity was 5, ranging from 4–7 (10 being the highest possible mark). The five articles assessed positively reached the external validity (item 1) and the statistic items (items 10 and 11). However, the internal validity punctuation was more heterogeneous. None of the evaluated studies met items 5, 6, and 7 related to the blinding process, difficult to fulfill in sport sciences.

**Table 1:** Risk of bias using the PEDro scale.

**Validity**	**External item**	**Internal items**								**Statistic items**		**TOTAL score**
**Study**	**1**	**2**	**3**	**4**	**5**	**6**	**7**	**8**	**9**	**10**	**11**	
Lee et al. (2018)	Yes	Yes	No	Yes	No	No	No	Yes	No	Yes	Yes	5
Zhou et al. (2018)	Yes	Yes	Yes	Yes	No	No	No	No	Yes	Yes	Yes	6
Dias Reis et al. (2017)	Yes	No	No	Yes	No	No	No	Yes	No	Yes	Yes	4
Mostarda et al. (2017)	Yes	Yes	Yes	Yes	No	No	No	Yes	Yes	Yes	Yes	7
Shin et al. (2016)	Yes	No	No	Yes	No	No	No	Yes	No	Yes	Yes	4
Niederer et al. (2013)	Yes	No	No	Yes	No	No	No	Yes	No	Yes	Yes	4

*Items: 1, eligibility criteria were specified; 2, subjects were randomly allocated to groups; 3, allocation was concealed; 4, the groups were similar at baseline; 5, there was blinding of all subjects; 6, there was blinding of all therapists; 7, there was blinding of all assessors; 8, measures of at least one key outcome were obtained from more than 85% of the subjects who were initially allocated to groups; 9, the intention-to-treat analysis was performed on all subjects who received the treatment; 10, the results of the between-group statistical comparisons are reported for at least one key outcome; 11, the study provides both point measures and measures of variability for at least one key outcome; total score, each satisfied item (except the first) contributes 1 point to the total score, yielding a PEDro scale score that can range from 0 to 10*.

### Participants Characteristics

The baseline information of the participants is reported in Table 2. The total sample size of the systematic review was 272, out of which 126 were included in the CG and 146 in the EG, and the sample was mainly composed of women. The ages ranged from 30 to 75 years, although most of the participants were older than 45 years of age. Different types of cancer were included in this study as follows: three interventions contained only patients with breast cancer (Shin et al., 2016; Dias Reis et al., 2017; Mostarda et al., 2017) and two incorporated various types of cancer such as colorectal, lung, breast, genital, gastrointestinal, and hematological (Niederer et al., 2013; Lee et al., 2018). One study contained only nasopharyngeal cancer (Zhou et al., 2018). The patients participated in the exercise program during the treatment of cancer drugs (i.e., chemotherapy, radiotherapy, and hormonotherapy) (Niederer et al., 2013; Shin et al., 2016; Zhou et al., 2018) or after finishing the treatments (Niederer et al., 2013; Dias Reis et al., 2017; Mostarda et al., 2017; Lee et al., 2018).

**Table 2:** Baseline characteristics of participants.

**Study**	**Design**	**Group**	***N*** **(% females)**	**Age**	**Cancer type**	**Cancer stage**	**Cancer treatment (type or timing)**
Lee et al. (2018)	RCT	CG	26 (84.6%)	30–39 (3.8%), 40–49 (19.2%), 50–59 (57.7%), 60–69 (11.5%), and 70–75 (7.7%)	Colorectal (11.5%), lung (3.8%), breast (69.2%), gynecological (7.7%), and other (7.7%)	I (42.3%), II (26.9%), III (26.9%), and none (3.8%)	After treatment: surgery, chemotherapy, radiotherapy, and target therapy
		EG	29 (86.2%)	30–39 (10.3%), 40–49 (24.1%), 50–59 (37.9%), 60–69 (17.2%), and 70–75 (10.3%)	Colorectal (6.9%), lung (3.4%), malignant lymphoma (3.4%), breast (62.1%), gynecological (6.9%), and other (17.2%)	I (24.1%), II (51.7%), III (20.7%), and none (3.4%)	
Zhou et al. (2018)	RCT	CG	57 (21.05%)	<30 (12.3%), 30–50 (64.9%), and >50 (22.8%)	Nasopharyngeal	III (40.3%), IV a/b (59.6%)	During treatment: chemotherapy and radiotherapy
		EG	57 (33.33%)	<30 (22.8%), 30–50 (59.6%), and >50 (17.5%)		III (35.1%), IV a/b (64.9%)	
Dias Reis et al. (2017)	Controlled trial	CG	9 (100%)	45 ± 7	Breast	I, II, and III	After treatment: hormonal therapy, chemotherapy, and radiotherapy
		EG	9 (100%)	48 ± 7.2			
Mostarda et al. (2017)	RCT	CG	9 (100%)	Range 30–59	Breast	I, II, and III	After treatment: hormonal therapy (33.3%), chemotherapy (27.8%), and radiotherapy (38.9%)
		EG	9 (100%)				
Shin et al. (2016)	Controlled trial	CG	10 (100%)	49.2 (range, 35–60)	Breast	0 (10%), I (20%), II (60%), and III (10%)	During treatment: anticancer treatment (80%) and radiotherapy (30%)
		EG	12 (100%)	46.3 (range, 35–66)	Breast	0 (8.3%), I (33.3%), II (50%), and III (8.3%)	During treatment: anticancer treatment (83.3%), radiotherapy (91.7%)
Niederer et al. (2013)	Controlled trial	CG	15 (60%)	61.6 ± 10.6	Each group: Gastrointestinal (20%), genital and breast (26.67%), diagnosis bronchial (13.33%), hematological (6.67%), and other (33.33%)	0, I, and II	Chemotherapy 12, Radiotherapy1, and Hormone therapy2
		EG (during)	15 (60%)	59.6 ± 9.4		0, I, and II	During treatment: Chemotherapy 14 and Radiotherapy1
		EG (after)	15 (60%)	60.7 ± 6.7		0, I, and II	After treatment

*RCT, randomized controlled trial; CG, control group; EG, exercise group*.

### Intervention Characteristics

The exercise interventions included in this review are detailed in Table 3. The length of the exercise program varied from 4 weeks (Mostarda et al., 2017) to 16 weeks (Niederer et al., 2013), with one intervention conducted during the entire chemotherapy cycle of 19 months (Zhou et al., 2018). Participants attended exercise sessions once per week (Lee et al., 2018), three times per week (Shin et al., 2016; Dias Reis et al., 2017; Mostarda et al., 2017), and five times per week (Zhou et al., 2018), whereas Niederer et al. (2013) suggested patients exercise 3–5 times per week. Together with the study of Zhou et al. (2018), their interventions were the only programs that included an unsupervised exercise element. Sessions lasted from 60 to 120 min (Niederer et al., 2013; Lee et al., 2018; Zhou et al., 2018), with a median of a 70-min exercise (Shin et al., 2016; Dias Reis et al., 2017; Mostarda et al., 2017).

**Table 3:** Characteristics of exercise interventions and heart rate variability (HRV) measurements.

**Study**	**Group**	**Length**	**Sessions duration**	**Weekly frequency**	**Exercise description**	**HRV measurement and analyses process**
Lee et al. (2018)	CG	12 weeks				Tool: CheckMyHeart Handel HRV ECG Duration: 5 min Position: seated or lying prone HRV variables: SDNN, TP, and HF Analysis information
	EG	12 weeks	120 min	1 time/week	Qigong: standing position, meditation, and leg massage.	
Zhou et al. (2018)	CG	During the chemotherapy of 19 months				Tool: ECG Duration:5 min Position: supine HRV variables: LF, HF, and LF/HF Analysis information: AcqKnowledge and Kubios and relative power and normalized units computation for frequency variables
	EG		60 min	5 times/week	Supervised or instructional video Tai Chi exercise: Warm up: 10 min Main part: 30 min Tai Chi exercise and 10 min of breathing and meditation Relaxation: 10 min	
Dias Reis et al. (2017)	CG	12 weeks				Tool: Tachogram Duration: 5 min Position: supine HRV variables: SDNN, RMSSD, LF, HF, and LF/HF Analysis information: beat-to-beat interval of iR–R, manually and automatic Kubios software filter, beat-by-beat sets were converted to equidistant time series and then applied the FFT
	EG	12 weeks	≈70 min	3 times/week	Cardiovascular training: 30 min cycle ergometer (60% VO_2max_) Resistance training: free weight exercise (hip flexion and extension, shoulder press, free squad, French triceps press, curved row General stretching: maintained each exercise 20–30 s	
Mostarda et al. (2017)	CG	4 weeks				Tool: Tachogram Duration: 5 min Position: NR HRV variables: SDNN, RMSSD LF, HF, LF/HF Analysis information: beat-to-beat interval of iR-R, manually and automatic Kubios software filter, FFT, and normalized units computation
	EG	4 weeks	70 min	3 times/week	Cardiovascular training: 30 min cycle ergometer (60% VO_2max_) Resistance training: squat, shoulder press, hip flexion, barbell bent over row, and French press	
Shin et al. (2016)	CG	8 weeks				Tool: CANS 3000 (wrists and ankles and electrodes) Duration: 5 min Position: sitting HRV variables: SDNN, RMSSD, LF, HF, LF/HF Analysis information: R–R interval (1/1,000 s)
	EG	8 weeks	70 min	3 times/week	Warm up: 10 min gymnastics and stretching (upper body) Main exercises: 40 min circuit exercises (Shaking while running in place, flank, running in place, squat, walking in place, crunch, step, lunge, running with open arms, back muscle exercise) Intensity control: RPE. Progression from 9 to 14 RPE Cool down: 15 min gymnastics and stretching	
Niederer et al. (2013)	CG	16 weeks				Tool: Polar S810 Duration: 5 min Position: supine HRV variables: TP, HF, LF Analysis information: FFT, Kubios HRV Analysis, Biosignal Analysis, and logarithmic values.
	EG (during)	16 weeks	NR (only recommen dations)	NR (only recommen dations)	Home-based training counseling (recommendation 3–5 times per week, 1 h, at 70–90% of individual anaerobic threshold, 13–14 RPE) and the opportunity to participate in a guided Nordic-Walking training (1 time/week)	
	EG (after)	16 weeks				

*CG, control group; EG, exercise group; NR, not reported; VO_2max_, maximum oxygen consumption; RPE, the rating of perceived exertion; SDNN, SD of the interbeat interval of normal sinus beats; RMSSD, root mean square of successive differences between normal heartbeats; LF, low-frequency band; HF, high-frequency band; LF/HF, Ratio of LF-to-HF; TP, Total Power; FFT, fast Fourier transform*.

Two exercise programs were based on oriental exercise techniques, such as Tai Chi (Zhou et al., 2018) and Qigong (Lee et al., 2018), including a meditation component. The remaining interventions involved cardiovascular and resistance training (Niederer et al., 2013; Shin et al., 2016; Dias Reis et al., 2017; Mostarda et al., 2017), including the program conducted by Niederer et al. (2013), where patients were counseled to exercise following the recommendations of the ACSM Roundtable on Exercise Guidelines for Cancer Survivors (Schmitz et al., 2010) and could participate in guided Nordic Walking sessions once per week. Dias Reis et al. (2017) and Mostarda et al. (2017) incorporated the cardiovascular cycling of 30 min and different free weight exercises, such as squats, shoulder press, hip flexion, or French press, among others (Dias Reis et al., 2017; Mostarda et al., 2017). In contrast, Shin et al. (2016) used circuit training to develop the sessions combining strength and cardiovascular exercises and using the rate of perceived exertion (RPE) to monitor and progress the intensity (Shin et al., 2016).

### HRV Results

Most of the studies reported the outcome results after treatment. However, one study (Lee et al., 2018) only reported the change from baseline. Therefore, it was excluded from this meta-analysis. Other than the study of Niederer et al. (2013), the outcomes of the EG after cancer treatment were extracted (Niederer et al., 2013). The results were then divided into time and frequency domains to present how exercise influences patients with cancer and survivors in these domains.

**Figure 2:**
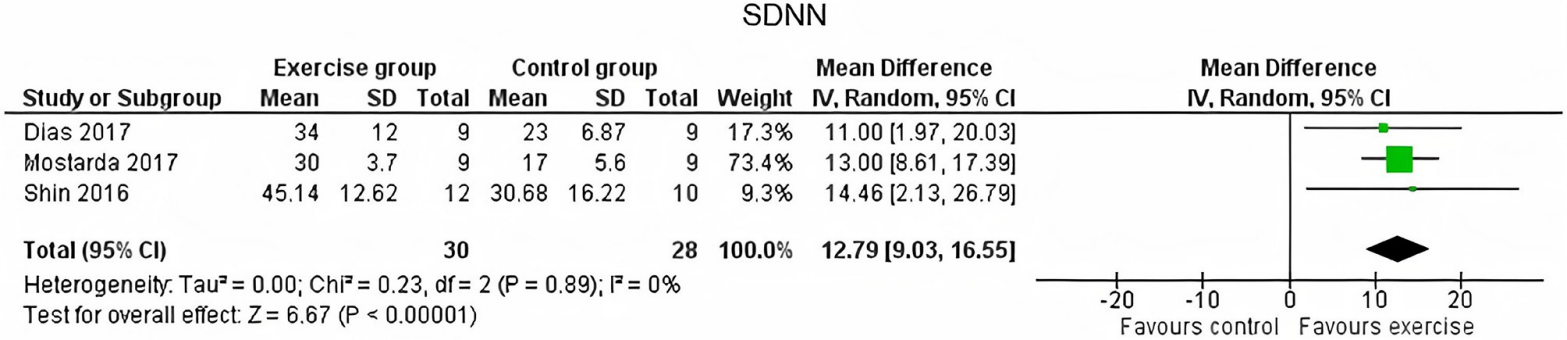
Effects of exercise in SDNN (standard deviation of the interbeat interval of normal sinus beats) heart rate variability measure.

**Figure 3:**
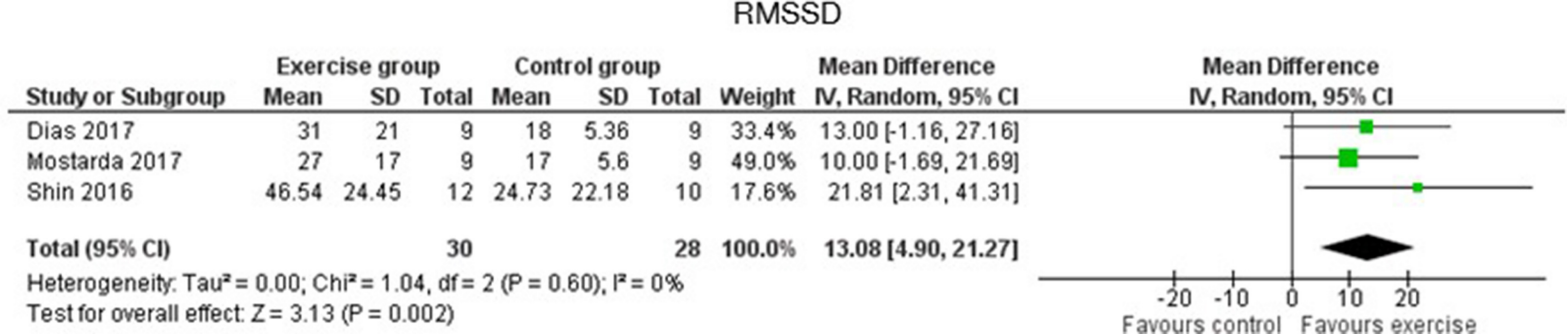
Effects of exercise in RMSSD (root mean square of successive differences between normal heartbeats) heart rate variability measure.

Regarding the HRV time-domain measure analysis, in SDNN EG, there were significant increases compared with the CG with a *p* < 0.00001 (MD of 12.79 and a 95% CI from 9.03 to 16.55) (Figure 2). Also, Lee et al. (2018) found significant differences between groups analyzing the change from baseline (*p* = 0.001) (Lee et al., 2018). Participants in the EG also reached higher increases on RMSSD compared to those in the CG (*p* = 0.002 with an MD of 13.08 ms and 95% CI from 4.90 to −21.27) (Figure 3).

**Figure 4:**
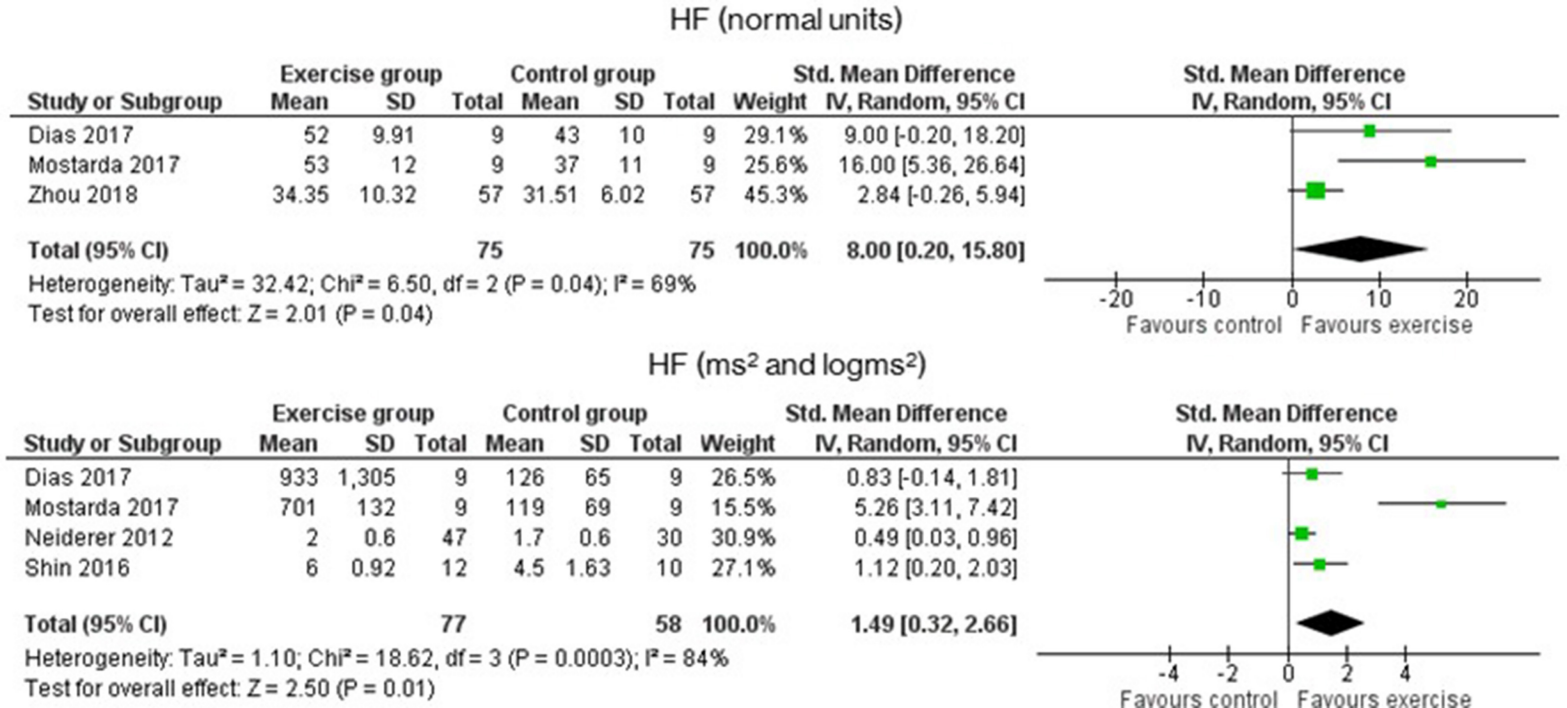
Effects of exercise in HF (High Frequency) heart rate variability measure expressed in absolute (ms^2^) and relative (n.u) power.

**Figure 5:**
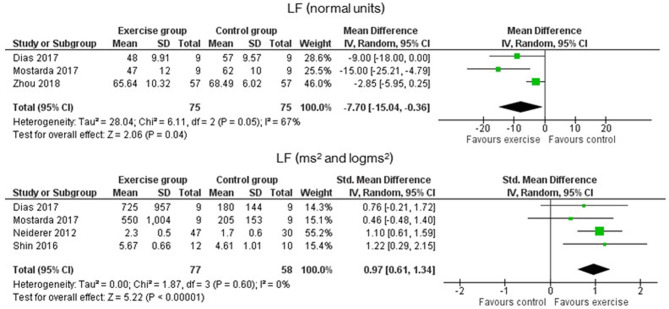
Effects of exercise in LF (Low Frequency) heart rate variability measure expressed in absolute (ms^2^) and relative (n.u) power.

**Figure 6:**
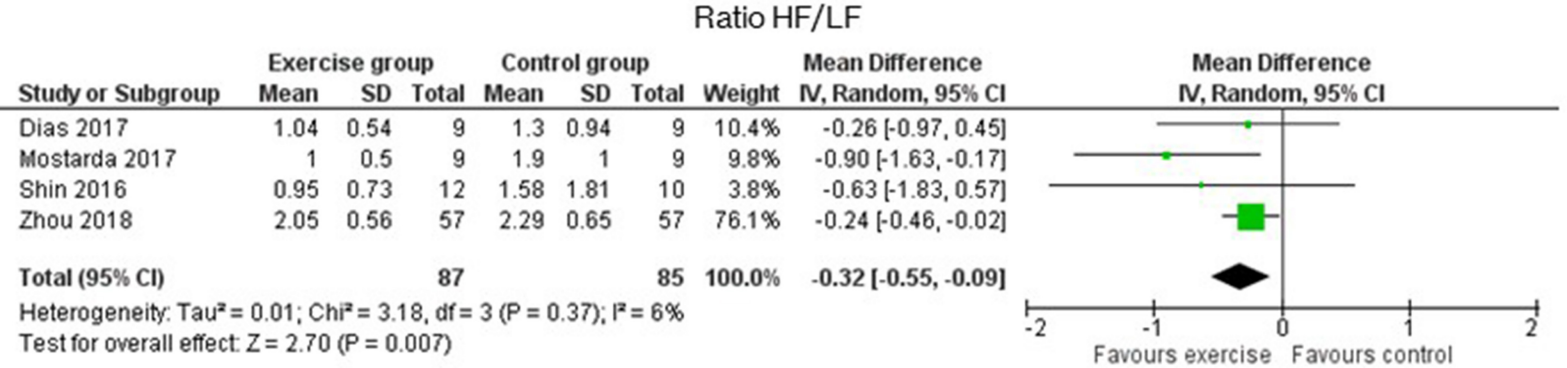
Effects of exercise in Ratio HF/LF (ratio of Low Frequency to High Frequency) heart rate variability measure.

As for the frequency-domain outcomes, the LF and HF results were obtained in SMD from the analysis of the absolute power (ms^2^ or log ms^2^), and that in MD, the relative power (n.u.). Figure 4 shows that LF was significantly increased in the EG compared to the CG in both analyses. In absolute power, results had a *p*-value of <0.0001 and an SMD of 0.97 with a 95% CI from 0.61 to 1.34, while in n.u., the *p*-value was 0.04 with an MD of −7.70 n.u. and a 95% CI from −15.04 to −0.36. The HF results, which are presented in Figure 5, of the EG in absolute power measures, were significantly higher than the CG outcomes (*p* = 0.001, and an SMD of 1.49 with a 95% CI from 0.32 to 2.66) and their effects on the relative power units (*p* = 0.04, and an MD of 8.00 n.u. with a 95% CI from 0.20 to 15.80). Moreover, Lee et al. (2018) stated that significant differences in the change from baseline reveal but not between groups (Lee et al., 2018). The ratio LF/HF showed significant differences between EG and CG with a *p*-value of 0.007 and an MD of −0.32 (95% CI from −0.55 to −0.09), as represented in Figure 6. Finally, the TP data was not analyzed by meta-analysis only two studies include the measure (Niederer et al., 2013; Lee et al., 2018). Niederer et al. (2013) stated the significant differences in the interaction between groups (i.e., exercise during treatment group, exercise after treatment group, and CG) and time (before and after intervention) with a *p*-value of 0.025. However, their *post-hoc* analysis showed high differences between after treatment group and the CG (*p* = 0.012). As for the results of Lee et al. (2018), the Qigong EG significantly increases TP with a *p*-value of 0.002 from baseline to after intervention.

## Discussion

This systematic review and meta-analysis aimed to evaluate the effects of exercise interventions in the HRV of cancer patients and survivors. The data obtained showed significant differences between the EG and the CG in all the variables analyzed as follows: SDNN, RMSSD, LF (ms^2^ and n.u.), HF (ms^2^ and n.u.), LF/HF ratio, and TP. Thus, exercise interventions may improve the autonomic control in patients with cancer and reduce the risk of autonomic dysfunction of participants. However, some specifications need to be considered due to the heterogeneity of the programs and the physiological implications of the variables analyzed.

Although we have included all the variables measured in the HRV, the physiological interpretation of some of these variables of ANS recording is controversial. Only RMSSD and HF have been proven to reflect the PNS activity to date (Shaffer and Ginsberg, 2017), whereas the reflection of SNS or PNS in SDNN, LF, and LF/HF is not clear. SDNN seems to measure the overall HRV with the contribution of sympathetic and parasympathetic modulation, but in the short-term resting recordings, the main source of the variation could be provided from the SNP (Shaffer and Ginsberg, 2017). The LF physiological interpretation is still not universally agreed since some researchers assume it as an index of cardiac sympathetic control (Reyes Del Paso et al., 2013), whereas more current literature state that it may principally reflect the baroreflex activity (Goldstein et al., 2011) or even being mainly determined by the PSN (Reyes Del Paso et al., 2013). However, it seems to depend on the band recording frequency having a possible SNS implication if it reaches 0.1 Hz (Shaffer and Ginsberg, 2017). Consequently, the physiological interpretation of the LF/HF ratio is also uncertain due to LF not being a pure SNS index (Goldstein et al., 2011; Shaffer and Ginsberg, 2017). With this in mind, the following discussion will be focused on the HF and RMSSD physiological values to argue the overall autonomic control effects.

In line with HRV parasympathetic activity variables of the CGs, several articles have revealed the effects of cancer treatments in RMSSD and HF and the overall measure of SDNN. Surgery, for instance, significantly reduces RMSSD and SDNN even 14 days post-op (Hansen et al., 2013), which is similar to what occurs after administration of a high dose of chemotherapy and its subsequent cardiotoxicity (Kloter et al., 2018). This ANS dysfunction reduces the release of catecholamine neurotransmitters, which could negatively influence the regulation of the tumor microenvironment (Hanns et al., 2019). Cancer decreases catecholamine production, with a concomitant rise in oxidative stress, inflammation, and cancer progression (Cole et al., 2015). Under normal conditions, the PNS could regulate the inflammatory response, but the decline of the vagal nerve activity produced by cancer may inhibit inflammatory regulation (Williams et al., 2019). In this way, in contrast to healthy controls with similar characteristics, patients with breast cancer have significantly lower RMSSD, HF, and SDNN values 1 year after treatment (Caro-Morán et al., 2016). Consequently, when comparing the HRV results with the normal values of healthy individuals, the differences are notable (Nunan et al., 2010). Thus, the role of PNS and its variance is so crucial in cancer prognosis that having a high HF power is positively correlated with survival in patients with advanced breast cancer (Giese-Davis et al., 2015).

**Figure 7:**
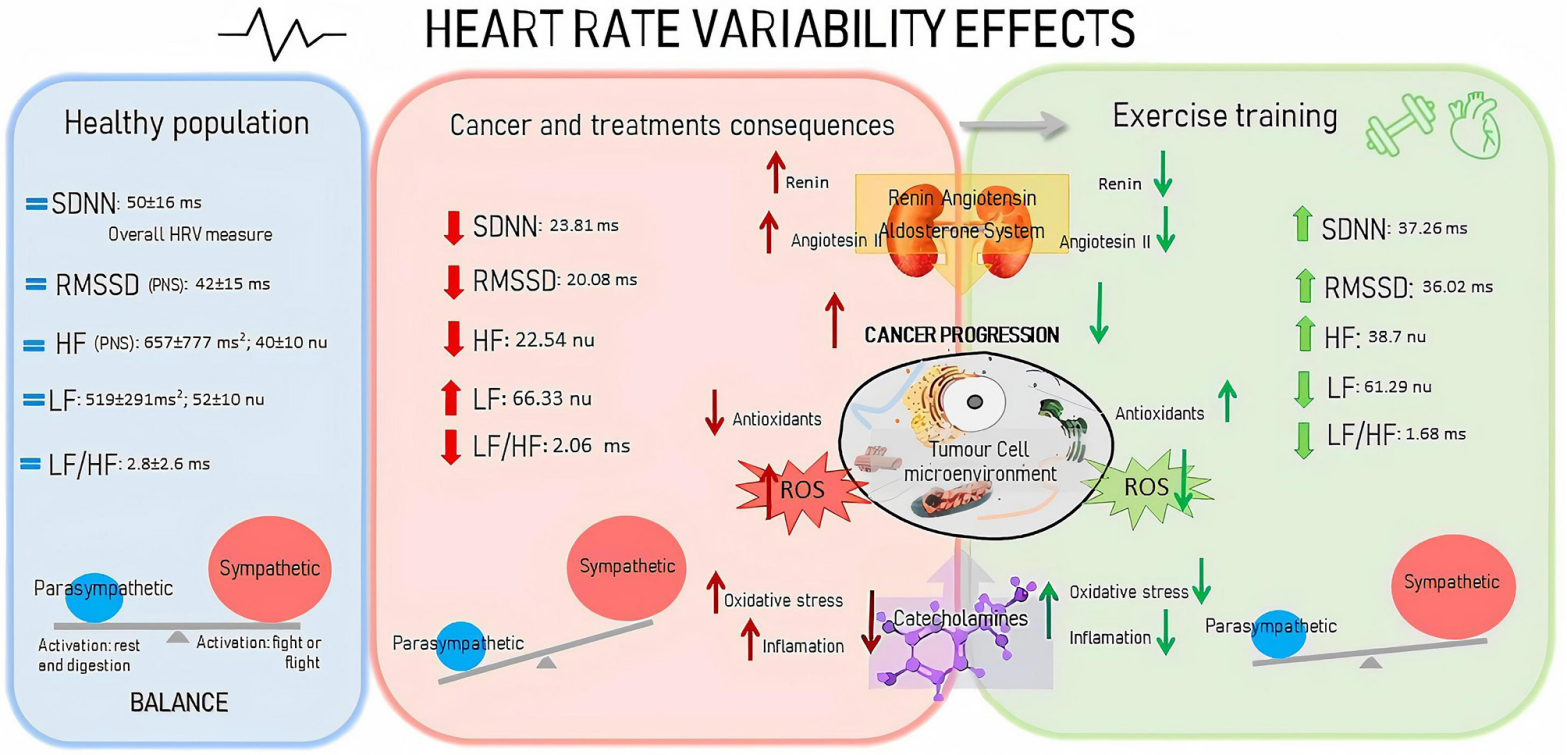
Explication of the changes in the heart rate variability of cancer patients and exercise training effects in comparation to healthy population data. Healthy population of HRV data from Nunan et al. (2010), cancer and treatments consequences and exercise effects data correspond to control and exercise group after intervention results of the current systematic review. References from the physiological changes in cancer patients and its exercise effects are reported in the systematic review discussion. SDNN, standard deviation of the interbeat interval of normal sinus beats; RMSSD, root mean square of successive RR interval measures; HF, high frequency; LF, low frequency; LF/HF, low-to-high frequency ratio; PNS, parasympathetic nervous system; ROS, reactive oxygen species.

Exercise seems to have a positive influence on the ANS of patients with cancer and its related physiological consequences, as shown in Figure 7, illustrates, in this case, the recovery to the normal values of HRV measures after the interventions. Exercise can induce the increase of catecholamines, which are commonly reduced due to cancer and lead to positive changes in tumor hypoxia, angiogenesis, metabolic stress, and cell immunity (Hojman et al., 2018) by the lactate production, according to the Warburg effect (San-Millán and Brooks, 2016). This would increase the parasympathetic responses and decrease the local oxidative stress and DNA damage, i.e., inflammatory reactions (De Couck et al., 2012). Consequently, the ability of cancer cells to form tumors in distinct tissues (Hojman et al., 2018) and the risk of developing metabolic abnormalities (Licht et al., 2010) related to poor cancer prognosis (De Couck et al., 2012) could be reduced. Moreover, exercise may also benefit patients by increasing the vagal nerve stimulation in the renin-angiotensin-aldosterone system (Miller and Arnold, 2019). When this occurs, there may be a reduction in the renin enzyme production (Cunha et al., 2016), with subsequent angiotensin II reduction, thereby affecting the cholinergic parasympathetic neurotransmission to the heart (Miller and Arnold, 2019). These mechanisms control angiogenesis, tumorigenesis, metastasis, and cellular proliferation (Munro et al., 2017). Few articles relate the effects of exercise in the renin-angiotensin-aldosterone of patients with cancer, but in other disease populations, it appears that exercise could prevent the increase of angiotensin-converting enzyme and plasma angiotensin II levels (Nunes-Silva et al., 2017) (Figure 7).

The type of exercise and the intensity of exercise could be the important factors to consider for the PNS activation. First, to increase catecholamine production, moderate- or high-intensity exercise is needed (Zouhal et al., 2008). Additionally, in HF values, no significant differences between the control and the Qigong group were found (Lee et al., 2018), and the Tai Chi intervention presented the lowest MDs results (Zhou et al., 2018), which could mean that the intensity performed was too low to impact the PNS activity. Accordingly, low responses were also shown in the intervention in the study of Niederer et al. (2013), where participants engaged in unsupervised physical activity without a structured program (36). Still, studies carried out with other target populations show that low-intensity exercise modalities such as Tai Chi increased the parasympathetic stimulation (Cole et al., 2016). However, a study with elderly women, which compared the effects of the autonomic modulation of Tai Chi and walking programs, found no significant HRV differences between the groups (Audette et al., 2006). Perhaps higher intensities may be needed to increase the muscle recruitment associated with the rise in circulating catecholamines (Spiering et al., 2008), which has been shown to decrease HRV (Zouhal et al., 2008). Moreover, the comparison between the effects of endurance training and resistance training on the autonomic modulation, measured by HRV, is still controversial in patients with chronic diseases (Boudet et al., 2017). Although endurance training seemed to be more effective in modifying the HRV activity in healthy populations, in patients with metabolic syndrome, the high-intensity resistance training together with endurance seemed to have greater decreases in the heart rate and greater increases in the VLF domain compared with moderate resistance training with endurance workout (Boudet et al., 2017). These improvements could be produced by the role of strength training in declining the inflammatory process, an aspect shared with cancer physiology (Gleeson et al., 2011). Besides, resistance training may be utilized to prevent or to regain the decline of HRV considering that sarcopenia is a significant predictor of toxicity and time to tumor progression (Prado et al., 2009). More investigation is needed to identify the type of optimal exercise and to analyze the physiological process of resistance exercise in HRV physiology.

Most of the sport science investigations performed about HRV measure the effects of acute doses of exercise during the practice and in the recovery phase. In cancer, the acute effects of exercise have been analyzed with HF and RMSSD measures during and after yoga practice obtaining significant HF alterations in all the positions performed except in meditation and post-resting (Mackenzie et al., 2014). Hence, in line with the previous literature, a higher muscle activation may be required during exercise to stimulate the vagal nerve activity. An intervention carried out with Tai Chi Qigong added that a minimum of 4 min of practice is required to achieve the effects in HF and LF (Fong et al., 2015). Moreover, HRV measures could provide an opportunity to record how participants have responded to training in the 12–24 h post-exercise session (Javaloyes et al., 2020). These HRV outcomes, usually measured by RMSSD, can guide to decide the intensity and volume of the following session of training (Kiviniemi et al., 2007; Javaloyes et al., 2020). Nevertheless, no investigations have been performed at present with patients with cancer.

The current meta-analysis and systematic review are the first to explore the effects of exercise programs on the HRV of patients with cancer and its survivors. Some limitations need to be mentioned. The total sample size was moderate at best, although interventions that involved all types of exercise were included. Consequently, the studies analyzed were heterogeneous, limiting the generalization of the results, but still provide a wider review of the types of interventions investigated in the field. Finally, only studies written in English or Spanish, indexed in PubMed or Web of Science and articles with a before and after HRV measure or changes from baseline outcomes were included.

## Conclusion

Exercise programs may lead to positive effects on the overall autonomic control, measured by HRV of patients with cancer and its survivors. This systematic review and meta-analysis show that exercise can increase SDNN (overall HRV), RMSSD, and HF (n.u. and ms^2^), reflecting the stimulation of PNS activity. Furthermore, significant differences between EG and CG were also found in the LF and the LF/HF ratio of HRV variables. Due to the low number of interventions performed on HRV, exercise, and cancer, no further conclusions can be made. Thus, future research is needed to contrast the findings and to provide more specific information about the type and intensity of exercise required to improve the overall autonomic control and to reduce the toxicity and future autonomic dysfunction of the patient with cancer.

## Data Availability Statement

The raw data supporting the conclusions of this article will be made available by the authors, without undue reservation.

## Author Contributions

AL-P, DC-M, and XM: conceptualization, resources, validation, and writing the original draft preparation. AL-P, DC-M, and AJ: methodology, writing the review, and editing. AL-P and DC-M: software, formal analysis, and data curation. GL, LH, and AJ: investigation. GL, LH, and XM: supervision. DC-M and AJ: project administration. All authors have read and approved the published version of the manuscript.

## Conflict of Interest

AL-P and AJ were employed by GO fit LAB-Ingesport. The remaining authors declare that the research was conducted in the absence of any commercial or financial relationships that could be construed as a potential conflict of interest.

## Publisher's Note

All claims expressed in this article are solely those of the authors and do not necessarily represent those of their affiliated organizations, or those of the publisher, the editors and the reviewers. Any product that may be evaluated in this article, or claim that may be made by its manufacturer, is not guaranteed or endorsed by the publisher.
